# Educational Material about Influenza Viruses

**DOI:** 10.3390/v11030231

**Published:** 2019-03-07

**Authors:** Seema S. Lakdawala, Naina Nair, Edward Hutchinson

**Affiliations:** 1 Department of Microbiology and Molecular Genetics, University of Pittsburgh, Pittsburgh, PA, 15219 USA; 2 School of Simulation and Visualisation, The Glasgow School of Art, 167 Renfrew Street, Glasgow G3 6RQ, UK; nainanair@gmail.com; 3 MRC-University of Glasgow Centre for Virus Research, 464 Bearsden Road, Glasgow G61 1QH, UK

**Keywords:** influenza virus, coloring, word find, STEM, educational material

## Abstract

To supplement a special edition of the journal *Viruses*, entitled “What’s New with Flu?”, influenza virus researchers have worked together to generate simple educational material to communicate their science to school students. Educational materials suitable for a range of ages are included, from coloring exercises for younger students through to explanations of cutting-edge science in straightforward language for older students. This article contains a handout with influenza facts, a coloring page, a glossary and word find and a connect-the-dots exercise explaining the ideas behind recently published scientific papers. Together, these materials are intended to make research on influenza viruses more accessible to students and teachers.

## 1. Introduction

Influenza viruses affect us all. Most people experience influenza as a mild but unpleasant illness which they suffer from repeatedly throughout their lives. Some people have worse experiences with the virus, and most of us will know someone who has been severely ill with influenza. As well as causing mild disease, influenza can cause serious illness including death, particularly in at-risk groups such as older adults, the immunocompromised, pregnant women and infants. Although severe disease only occurs in a small minority of influenza cases, the large number of people infected with influenza each year means that influenza is a major healthcare challenge in countries throughout the world.

Understanding influenza should therefore be an important part of our education about health and disease. Knowing what we can do to minimize the risk of spreading influenza, understanding the benefits to ourselves and others of getting influenza vaccinations, and learning that the major investments society has made in basic science are now resulting in the development of new drugs to protect us against influenza, should all be public knowledge. The threat of new influenza pandemics is much discussed, and so it is important that we also learn why influenza pandemics of the past were so disruptive, and why scientists now view highly-pathogenic avian viruses such as the H5N1 and H7N9 strains of influenza with such concern.

**Figure 1:**
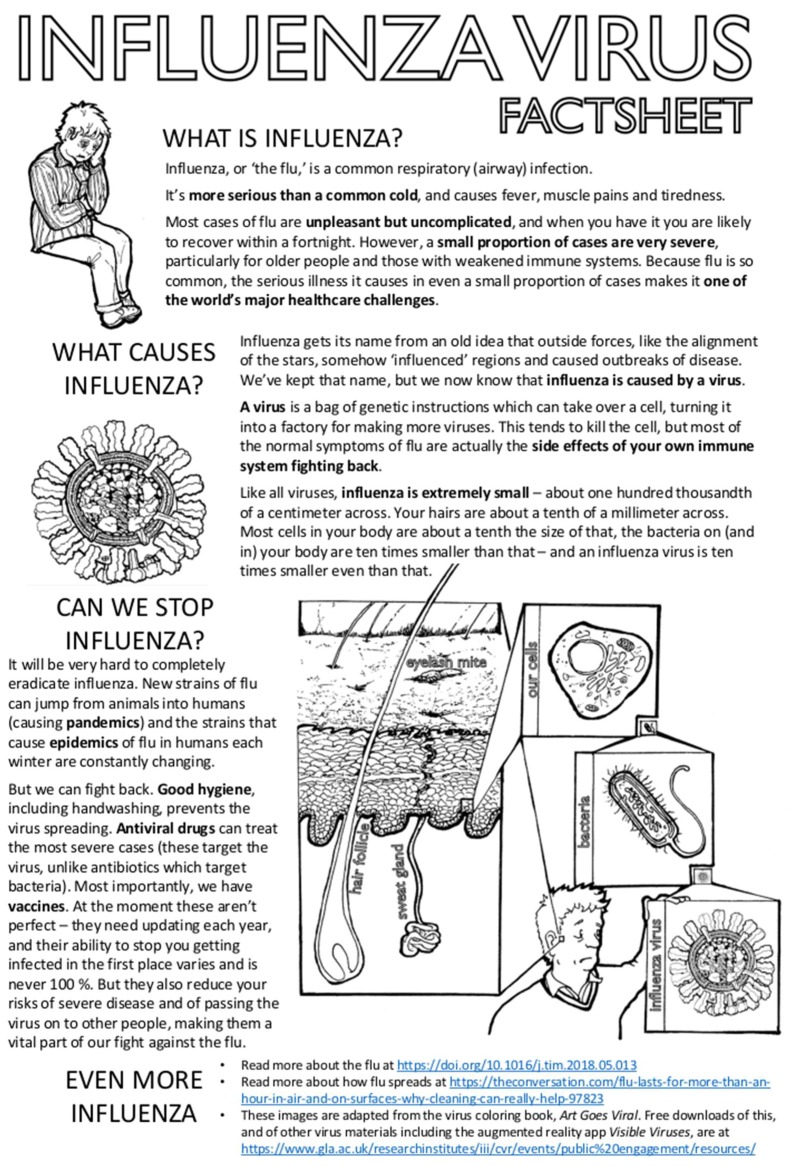
Influenza virus factsheet. A collection of basic facts about influenza viruses with links to further resources (a mini-review at https://doi.org/10.1016/j.tim.2018.05.013, a short article at https://theconversation.com/flu-lasts-for-more-than-an-hour-in-air-and-on-surfaces-why-cleaning-can-really-help-97823 and free educational resources at https://www.gla.ac.uk/researchinstitutes/iii/cvr/events/public%20engagement/resources/).

**Figure 2:**
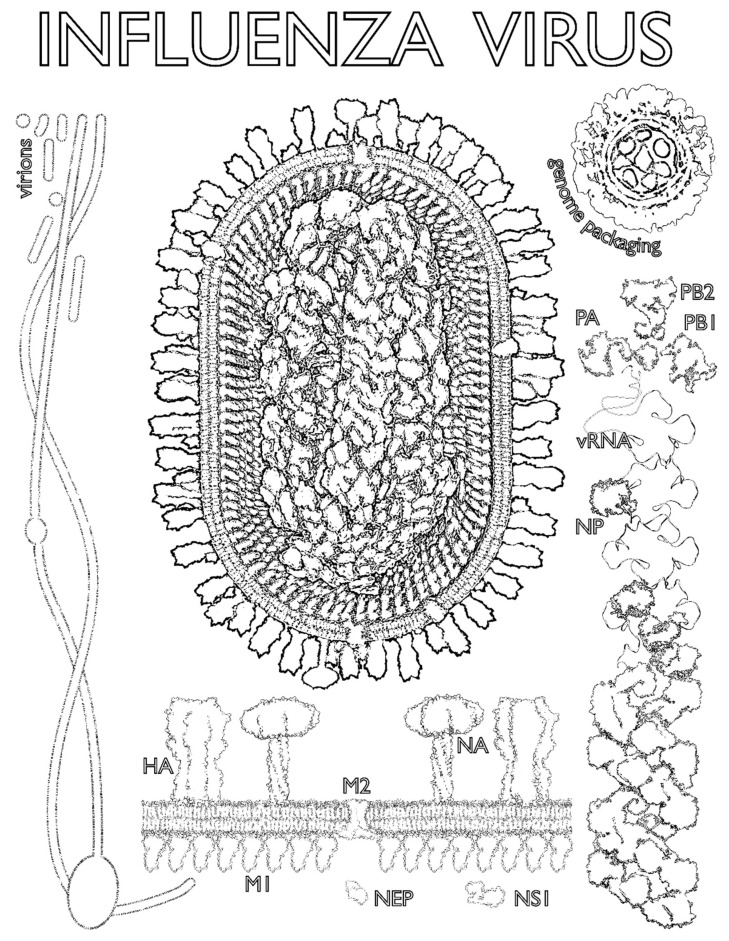
Influenza virus coloring page. A detailed composite image of an influenza virion (virus particle) and its component parts (below and right) with low-resolution images of the packaging of the viral genome (top right) and the filamentous virions seen in natural infections (left). PB1, PB2: basic polymerase subunits 1 and 2; PA: acidic polymerase subunit; HA: hemagglutinin; NP: nucleoprotein; NA: neuraminidase; M1, M2: matrix proteins 1 and 2; NS1: non-structural protein 1; NEP: nuclear export protein; vRNA: viral ribonucleic acid (genome). See text for further details.

**Figure 3:**
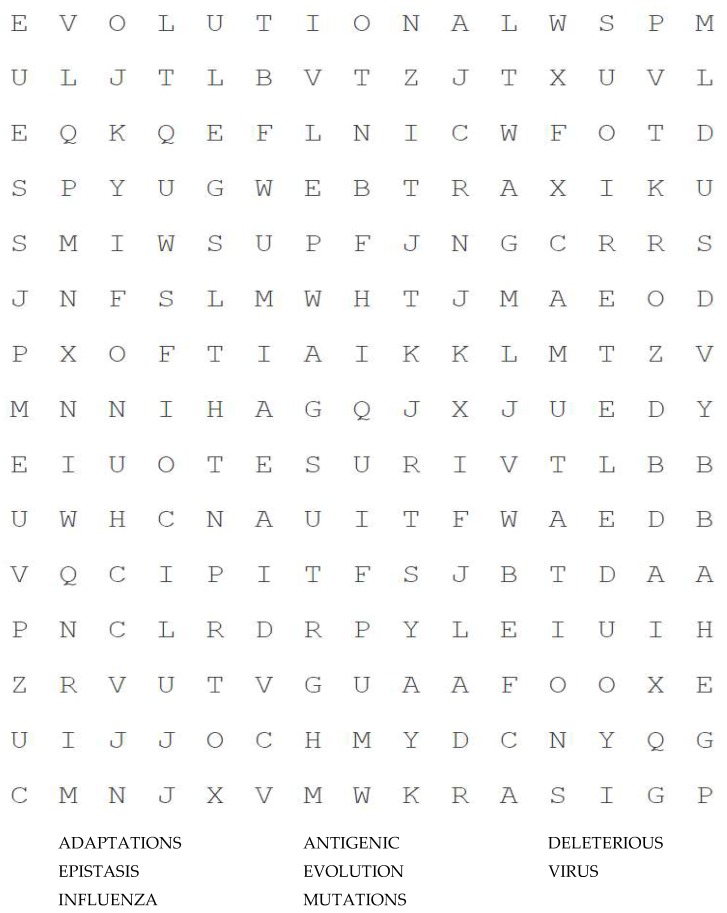
Viral epigenetics word find. See text for definitions.

**Figure 4:**
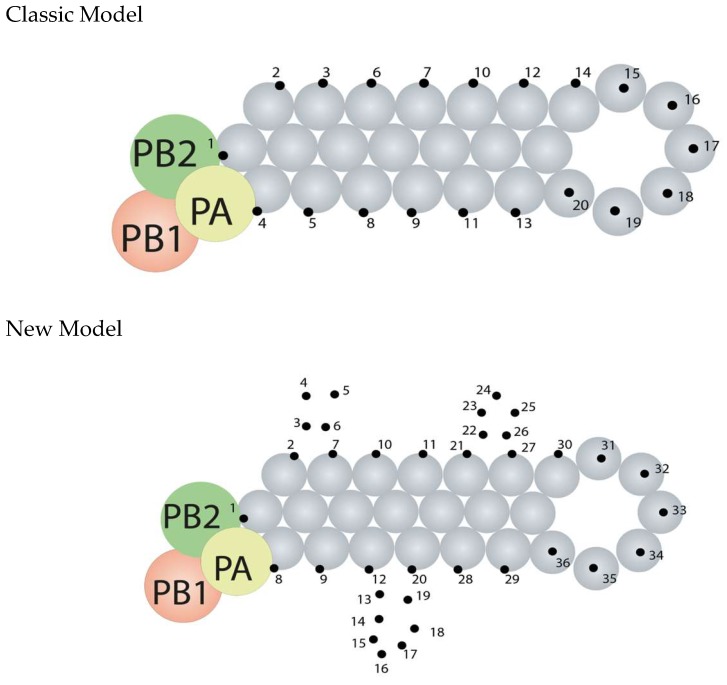
Two models for the binding of vRNA to proteins when assembling the influenza virus genome. See text for details.

This article is written by scientists for school students and educators, as a supplement to a collection of articles written by scientists for other scientists. Sharing our latest findings in articles is a crucial part of making progress in science, but detailed explanations of cutting-edge research are not usually very accessible to non-specialist readers. Like most scientists, we want to be able to share our excitement about what we do and make the topics we work on more accessible to the public who fund our work and who will, we hope, benefit from it in the future. There are many routes for doing this. One of them was to add this article to a collection of formal scientific articles, to make their content more accessible to the next generation of students. This article begins with a factsheet about influenza (Figure 1) and then moves on to three exercises directly inspired by recent and ongoing research in the authors’ laboratories (Figure 2, Figure 3 and Figure 4). We hope that it will be of use and of interest.

## 2. Influenza Virus Coloring Page

### 2.1. What Does an Influenza Virus Look Like?

One of the defining features of viruses (or, strictly speaking, of ‘virions’ or ‘virus particles’—the structures that wrap up a virus’ genes and carry them out of an infected cell and on to infect another cell) is that they are extremely small (Figure 1). Virions are smaller, in most cases, than the wavelength of visible light, which creates a challenge: how can we determine a virion’s structure? In other words, how do we work out how the basic building blocks of a virion physically fit together in enough detail to understand what makes it infectious? Two different approaches can be used for this.

One approach can be used for virions which form according to a very fixed pattern. Viruses of this sort, such as polioviruses, Zika viruses or adenoviruses, produce beautiful, geometrically regular virions. These are so similar to each other that an extremely detailed picture of an ‘average’ virion can be built up by combining low quality data from large numbers of individual virions. Virions can either be combined physically into a crystal which is then used to scatter X-rays, or many hundreds of them can be imaged individually using an electron microscope, creating data which are then combined using a computer.

Unfortunately, this approach does not work for viruses such as the influenza virus, the virions of which are variable in structure. While geometrically regular virions behave like carefully assembled boxes, with everything locked into its proper place, the flexible shells of influenza virions mean that they behave more like hastily packed bags, scooping up material from the infected cell. No two influenza virions are identical, and so we cannot build up a clear view of them by taking an average image. 

Instead, we can try and build up a composite picture by combining studies of different features of the virion. Figure 2 shows images of the influenza virion created by combining low-resolution images of the virion’s overall shape, a detailed ‘parts list’ of its components, and high-resolution images of those individual components. These include the bag-like membrane that surrounds the virion, reinforced beneath with a shell made of matrix protein 1 (M1) and studded with a matrix protein 2 (M2), which is a pore that lets the virion sense when it is inside a new cell. The membrane is decorated with spikes of hemagglutinin (HA) and neuraminidase (NA), the proteins that get the virion into and out of cells, respectively, and which are the main targets for the immune system (and, therefore, a way of categorizing types of influenza—H5N1, H3N2, etc.). Inside the virion, segments of the viral RNA genome (vRNA) are wrapped around proteins into rod-like complexes (the polymerase proteins PB2, PB1, PA and the scaffold-like nucleoprotein NP; shown bottom right and described further in Section 4 below), eight of which bundle together to allow packaging of a complete genome (shown in cross-section at top right and side-on in the main image). In order to be infectious, the virion needs to package all eight segments of the viral genome, but there is also room for it to take up other viral proteins such as the nuclear export protein (NEP) and non-structural protein 1 (NS1), as well as material from the host cell—when you sneeze, there are bits of you in every particle of flu. Finally, although most laboratory strains of influenza produce virions shaped like spheres or beans, virions in natural infections show extreme variations in form, including very long filamentous virions (left).

### 2.2. How Did We Produce This Image?

Figure 2 summarizes a large amount of work, some of which has been published and some is ongoing. The ‘parts list’ was obtained by methods including mass spectrometry, which provides the ratio of components but not their structures. The structures were obtained through studies using X-ray crystallography, electron microscopy and nuclear magnetic resonance. They were downloaded from the Protein Databank (PDB, www.rcsb.org; this site also includes many excellent educational resources) using the following PDB numbers: PB2, PB1 and PA (PDB 4WSB [1]), HA (PDB 1RU7 [2]), NP (PDB 2IQH [3]), NA (head PDB 3BEQ and stem PDB 1GCL [4,5], as [6]), M1 (PDB 1EA3 [7]), M2 (PDB 2L0J [8]), NS1 (PDB 4OPH [9]) and the RNP helix (PDB 4BBL [10]). The structures of NEP and parts of M1 and M2 have not been determined experimentally and so were predicted from their protein sequence using an algorithm called QUARK [11,12]. The membrane structure was simulated using an approach called molecular dynamics [13]. The overall shapes of the virions were determined at low resolution by electron microscopy [14,15]. Composite images were assembled using a range of software: PyMOL (Schrödinger, LLC), UCSF Chimera [16], QuteMol [17], Autodesk 3ds Max 2017 (Autodesk) with the Molecular Maya 2016 plugin, GIMP (http://gimp.org) and Inkscape (https://inkscape.org).

## 3. Viral Epigenetics Word Find Definitions

In this special edition of *Viruses* is an article which discusses the literature on the evolution of influenza viruses in the human population. The following words will help you understand some of the concepts discussed in this article, “*Mutation and Epistasis in Influenza Virus Evolution*” [18]. Once you know what they mean, try finding them in the word find (Figure 3).

**ADAPTATIONS**—are changes that increase a virus’ fitness in a particular environment. In this case, fitness refers to a virus’ ability to survive, reproduce, and spread. Therefore, an adaptation is something that improves a virus’ ability to reproduce within a host and transmit between them.

**ANTIGENIC**—antigens are molecules that are recognized by our immune system. In the case of influenza, the surface proteins (HA and NA—see Figure 2) are “antigenic,” as they are the proteins most commonly recognized by the immune system, and therefore the main components which are targeted by vaccines.

**DELETERIOUS**—in this case is something that is harmful to the virus. A deleterious mutation generally disrupts a fundamental function of the virus. Deleterious mutations are typically removed by natural selection. For a non-viral example, a mutation that resulted in a three-legged deer would be deleterious. It would be culled from the deer population as three legged deer would not be able to outrun predators and would not pass on the mutation to their offspring.

**EPISTASIS**—refers to how two different mutations interact to give a certain trait. A classic example of epistasis is fur color in animals, which is determined by several genes and combinations of mutations.

**EVOLUTION**—refers to the change in the characteristics of a species or population over time.

**INFLUENZA**—is a respiratory illness caused by a group of viruses, called influenza viruses. Common symptoms of influenza include fever, body aches, runny nose, cough and trouble breathing. It is commonly referred to as “the flu.”

**MUTATIONS**—are changes in the genes of a virus or organism. Since genes are the ‘instructions’ for a virus, mutations can often change the characteristics of a virus.

**VIRUS**—is a microscopic microbe that can only reproduce within another organism.

## 4. Influenza Virus Genome

The influenza virus genome is made of eight single-stranded RNA segments (Figure 2). The viral nucleoprotein (NP; depicted as gray beads below) is a virally encoded RNA-binding protein, which acts as a helical ‘scaffold’ for neatly storing the viral genome. The viral polymerase (made up of viral proteins PB2, PB1, and PA), is an enzyme complex which makes new copies of viral RNA (vRNA; genome) and messanger RNA (mRNA). The viral polymerase and NP are required for viral genome replication as well as the packaging of all eight genome segments into a single virion (Figure 2). Recent data [19,20], including a paper in this special edition of *Viruses*, have reshaped our knowledge of how NP binds to vRNA and have changed our model of the influenza virus genome’s structure. Classically, NP was thought to bind vRNA all the way along its length: imagine tightly strung beads on a string. However, ‘deep sequencing’ technologies (new and powerful methods of genome sequencing) have now been used to identify the regions of vRNA which are bound by NP. This revealed that, in fact, NP binds RNA in a non-uniform manner, leaving regions of vRNA exposed. 

To visualize this, connect the dots in Figure 4, starting with dot 1, to reveal vRNA in the classic model and then in the revised model. Notice the exposed regions of vRNA in the ‘new model’ of the viral genome. Because they are exposed, these regions of vRNA might bind to other genome segments to co-ordinate the packaging of all eight segments into a virion, or they might interact with RNA-binding proteins produced by the host cell. Now that we have a clearer model of the structure of the viral genome, we can plan further research to try and find out what this exposed vRNA is doing during influenza virus replication.

## 5. Conclusion

Effective science communication comes from many sources, including professional science communicators, journalists and educators. Ideally, it should also come from scientists themselves. While the resources in this article provide only a brief glimpse of the field of influenza research, we hope that they make biomedical research of the sort published in this special edition of *Viruses* more accessible, that they make the scientists who carry it out more approachable, and that they communicate at least some of the excitement we feel about being able to conduct scientific research on influenza viruses.
